# Supplementary material for: Sodium molybdate does not inhibit sulfate-reducing bacteria but increases shell growth in the Pacific oyster Magallana gigas
Source: PLoS One. 2022 Feb 9;17(2):e0262939. doi: 10.1371/journal.pone.0262939 (PMC8827440; doi:10.1371/journal.pone.0262939)
Supplement: Attachment — Submitted filename: Gold-Response-to-reviewer.pdf [file pone.0262939.s012.pdf]

Response to Reviewers

Editor Comments:

Line 188, add the reference for the primers used.

Change made, Parada reference added.

Take also the time to review the references as some of them are not in the same format (e.g., capitalization of the title, journal abbreviation, italics in scientific names).

The references have been reviewed and fixed.

Reviewer #1: Overall Comments:

The main goal of this manuscript was to characterize the effects of sodium molybdate on the Pacific oyster M.gigas based on the fact that sodium molybdate would competitively replace sulfate ions, inhibiting activity by sulfate-reducing bacteria (SRBs) and potentially leading to decreased oyster growth due to the influence of these bacteria on calcification. But this manuscript lacks a critical component when it comes to sulfate-reducing bacteria and the experimental setup.

The experimental setup was not oxygen-deprived, and often sulfate-reducing prokaryotes thrive in anaerobic environments. This is evident from the presence of SRBs in low abundances in the system. Since SRBs are present in such low abundances in the system, it is hard to reach any conclusion.

The reviewer brings up an important caveat regarding the role of SRBs in animal biomineralization. However, there is ample evidence that SRBs are capable of precipitating calcium carbonate in aerobic environments, including oxygenated sediments and microbial mats (e.g. Visscher et al. 2000; Jørgensen BB 1994; Canfield D & Des Marais DJ 1991; Cypionka et al. 2000). There are also plenty of opportunities for anaerobic microenvironments to develop in the oyster shell, either in fluid-filled spaces within the shell or during times of oyster valve closure (Vermeij 2013; 2020). Our goal was to study the role of SRBs in shell formation under natural conditions, so artificially creating anaerobic environments would not make sense in this context. We have added a new paragraph in the introduction to bring up these relevant points and references.

Microbiome harvesting, DNA extractions, and sequencing depth will have a huge impact on these lower abundance data. The changes in different taxa can also be contributed by the presence of multiple copies of the 16S rRNA gene in a bacterium which was not accounted for in this study.

We acknowledge that 16S rRNA gene copy number variability could cause certain microbial taxa to appear more abundant than is actually the case. This is a challenge for all microbiome studies that use the amplicon sequencing approach. How to correct for gene copy number remains an open question and tools developed for this purpose have been unreliable thus far (e.g. Louca et al., 2018; Stark et al., 2021). For these reasons, we chose not to correct for 16S rRNA copy number in this study. We added text and references to the Methods subsection “Microbiome Analysis and Visualization” to note this concern and justify our decision to not perform corrections.

Other critical questions are
1) Do all SRBs promote calcification?

There is compelling evidence that SRBs as a group can promote calcification, but there is uncertainty in the extent to which particular clades do so, the environments in which they can do it, and their role in animal calcification. Our new paragraph in the introduction includes these points.

2) Is there any other microbial population in the system which can affect oyster growth or promote calcification?

There are almost certainly other microbial taxa that affect oyster calcification. The entire microbial community dictates the alkalinity of the microenvironment and the subsequent probability that minerals will precipitate. While our focus in this project was on SRBs, we have added this caveat to the discussion.

Other microbial populations which were abundant in the system were not reported/discussed in the manuscript. Those microbial populations can also play a role in promoting the growth of Oysters. So, the antagonistic behavior of sodium molybdate could have been countered by the activity of other microbial populations.

We do provide a summary report of the microbial clades that exhibit significant differences between treatments (see section “Patterns and differences in microbial community composition”, Figure 7, and Table S6). As mentioned in the previous response, it is certainly possible that some of these non-SRB clades play a role in calcification. While we have no direct evidence of that in this study, we allude to the possibility in the discussion (see our response to the previous point). We are also preparing a follow-up study that will allow us to compare the microbial community in our experimental oysters to animals in other environmental conditions that produce similar changes in shell structure. That has the potential to reveal other candidate microbes that are broadly associated with shell growth. We appreciate the Reviewer’s suggestion for this analysis.

I feel that the starting microbial community will also be a determining factor for supporting/contradicting the hypothesis.

We do have microbiome data from pre-settlement larvae. However, the microbial community in larvae is dramatically different from post-settlement oysters, and provides little information on “starting” conditions relevant to the interpretation of the experiment. Transcriptome and 16S data from the larval treatment are publicly available through our NCBI Bioproject, and we anticipate discussing this data in a follow-up manuscript.

Despite these limitations, we feel the control population provides sufficient insight for us to test the impact of sodium molybdate on the oyster microbial community. If the reviewer wishes to expand on how they think our hypotheses could be strengthened with information from the “starting” community, we would be happy to consider it.

Specific comments:
Line 49: “A previous study showed ……” in place of “[21] showed”. Then the reference should be added at the end of the sentence.

Change made.

Line 61: 1500 L

Change made.

Line 78: 20 μm

Change made.

Line 82: What were the criteria for selection based on egg quality?

Text added: Eggs were considered good quality if they had a regular, round shape with a plump appearance, as opposed to ovate or wrinkly.

Line 145: 35 μm

Change made.

Line 181: Elaborate the method heading

Changed to “16S rRNA Sample Preparation and Sequencing”

Line 181: S will be in uppercase for 16S; here and in other places.

Change made.

Line 205: Add reference for Decontam’s prevalence method.

Reference added: Davis NM, Proctor D, Holmes SP, Relman DA, Callahan BJ (2017). “Simple statistical identification and removal of contaminant sequences in marker-gene and metagenomics data.” bioRxiv, 221499. doi: 10.1101/221499.

Line 210: >25,000 ASVs or a total of >25,000 ASV abundances?

Text changed from “but all remaining samples had >25,000 ASVs” to “but each remaining sample had >25,000 ASVs”

Line 223: Bray-Curtis dissimilarity

Change made.

Line 532: What does concentration of bacteria mean? Will it be abundance?

Response: “concentration” changed to “abundance”
